# Survival benefits of antipredator training in captive-reared salmon: impact of behaviour, health, and genetics

**DOI:** 10.1007/s00442-025-05821-1

**Published:** 2025-11-06

**Authors:** Ines Klemme, Pekka Hyvärinen, Anssi Karvonen

**Affiliations:** 1 Natural Resources Institute Finland (Luke), Migratory Fish and Regulated Rivers, Paltamo, Finland; 2 Department of Biological and Environmental Science, University of Jyvaskyla, Jyvaskyla, Finland

**Keywords:** Cognition, Fisheries management, Hybridisation, Infection, Salmon conservation

## Abstract

Releasing captive-reared animals into the wild is a common population management practise, but their inexperience with predators often leads to high post-release mortality. Although captive animals can be trained to recognize and respond to predatory cues, the post-release survival benefits of this method remain uncertain. Additionally, how factors related to captive breeding and rearing—such as hybridization and disease, which can affect learning and memory—influence the effectiveness of antipredator training has not been investigated. We conducted two experiments with Atlantic salmon (*Salmo salar*), during which they first underwent antipredator training via paired exposure to predator cues (Northern pike, *Esox lucius*) and conspecific alarm cues, followed by release into semi-natural streams for predation trials with live pike. The first experiment focused on post-release behaviours and demonstrated innate predator avoidance that was not enhanced by the training. In the second experiment, genetic background (purebred versus hybrid crosses) and parasite infection status (trematode eye fluke, *Diplostomum pseudospathaceum*) of the salmon were manipulated to assess their effects on antipredator learning. This experiment demonstrated a clear survival benefit from the training, which was not influenced by either the genetic background or infection. The variation in effectiveness of the antipredator training between the experiments may be attributed to different experimental environments and developmental stages of the salmon. Overall, our findings suggest that antipredator training conducted under specific conditions enhances post-release survival of captive-reared salmon, providing valuable insights for reintroduction and population augmentation programmes.

## Introduction

Population management strategies, such as the supplementation with captive-reared animals, are commonly employed to mitigate ongoing population declines. However, these efforts often have low success because animals raised in artificial environments are ill-equipped to cope with the challenges encountered in the wild (Olla et al. 1998; Brown and Laland 2001; Mathews et al. 2005; Jule et al. 2008). One reason is that captive breeding can alter selective pressures, a process referred to as hatchery or captivity-induced selection, which may result in maladaptive traits in natural habitats, such as increased susceptibility to predation (Huntingford 2004). Additionally, the stimulus-poor environments typical of many rearing facilities can hinder the development of key survival behaviours, such as predator avoidance (Huntingford 2004). As a result, captive-reared animals often fail to detect or appropriately respond to predators, leading to high rates of post-release predation (Berejikian 1995; Henderson and Letcher 2003; Moseby et al. 2011). A commonly proposed method to improve post-release survival is life-skill training, including the learning of predator information (Brown and Laland 2001). While such antipredator training has shown promise in laboratory settings, its effectiveness under natural conditions remains largely untested, leaving key questions about its practical application (Brown et al. 2013; Edwards et al. 2021; Zhu et al. 2023).

While antipredator behaviour has a strong innate component, experience with predators can trigger its expression, enhance its effectiveness, and provide information about novel predators (Griffin et al. 2000). Antipredator training is typically based on associative learning and involves paired exposure to predator cues and an aversive stimulus, conditioning a fear response (Griffin et al. 2000). Often, the aversive stimuli are alarm cues, such as auditory calls or chemical substances released from conspecifics (Griffin et al. 2000). Several laboratory studies have demonstrated improved predator avoidance following training (Rowell et al. 2020; reviewed in Edwards et al. 2021; Zhu et al. 2023), but these controlled environments do not capture the complexities associated with transitioning to the wild. Both learning and memory are highly plastic processes that are influenced by various environmental factors (Brown et al. 2013), highlighting the need for validating the laboratory findings in natural conditions.

In addition to environmental influences, cognitive performance can also be affected by inherent factors, such as health status and genetic background. For example, parasitic infections can negatively influence learning abilities in a range of taxa (e.g. Gegear et al. 2006; Jukes et al. 2002; Kavaliers et al. 1995; Templé and Richard 2015). In rearing facilities, unnaturally high population densities often result in increased disease susceptibility and spread, potentially compromising the success of antipredator training. Moreover, conservation-focused breeding programmes increasingly employ genetic management strategies, such as hybridisation to enhance genetic variation (Ralls et al. 2018; Chan et al. 2019; Hoffmann et al. 2021). However, hybridisation can also lead to maladaptive learning through genetic incompatibility or intermediate cognitive phenotypes that do not match either parental environment (Rice and McQuillan 2018). On the other hand, inbreeding has negative impacts on cognitive performance (Gavriilidi and Linden 2024), suggesting that alleviating inbreeding effects through hybridisation could enhance the effectiveness of antipredator training. Despite these potential benefits and challenges, the impact of infection and hybridisation on antipredator training remains unexplored.

The aims of this study were twofold: to explore the impact of antipredator training on the post-release survival of hatchery-reared Atlantic salmon (*Salmo salar*), and to investigate whether possible survival benefits depend on health status and genetic background of the fish. Salmon populations are endangered worldwide due to overexploitation, habitat destruction and climate change (ICES 2017). To support these declining populations, hatchery-raised individuals are regularly introduced, but the success of these actions is often deteriorated by high mortality rates (Aprahamian et al. 2003; Naish et al. 2007; ICES 2023). We conducted two experiments, in which salmon underwent antipredator training and were subsequently released to semi-natural streams with live predators (Northern pike, *Esox lucius*). Experiment 1 focused on antipredator behaviours and their relationship with survival. Experiment 2 investigated whether parasite infection and hybridisation between distinct salmon populations affect the post-release success of antipredator training. Specifically, salmon were exposed to the fluke *Diplostomum pseudospathaceum*, which reduces the eyesight of the fish by causing a condition known as parasitic cataracts. In addition, salmon originated either from a highly inbred landlocked population, a more diverse anadromous population, or their hybrids. Together, these studies improve our understanding of the effectiveness of antipredator training as part of reintroduction and population augmentation programmes.

## Methods

### Animal origin

Atlantic salmon (*Salmo salar*) used in Experiment 1 originated from a broodstock (population Tornio), maintained by the Natural Resource Institute Finland (LUKE). Individuals were tagged at ten months of age using 12 mm HDX passive integrated transponders (PIT), which were injected in the body cavity under mild anaesthesia (Benzocaine 40 mg L^−1^). After tagging, 400 fish were transported to LUKE’s Kainuu Fisheries Research Station (www.kfrs.fi), a flow-through experimental facility. They were maintained in replicated semi-natural outdoor streams (40 m^2^, without predators) for 3.5 months, and subsequently in replicated indoor tanks (15 m^2^) for 12 months. Two weeks before the experiment commenced, a total of 144 two-year-old smolts (juvenile migratory life stage) were randomly selected and measured for length (mean ± SD, 199.6 ± 19.4 mm) and mass (72.5 ± 22.2 g).

Parental fish for Experiment 2 originated from two populations: Saimaa landlocked salmon (LS, *Salmo salar* m. *sebago*) captured from the river Pielisjoki (62°35'N, 29°43'E), which drains into Lake Saimaa in Finland, and anadromous Atlantic salmon (AS) from a broodstock that originates from the River Neva (59°56'N, 30°16'E), which flows into the Gulf of Finland in the Baltic Sea. The LS population is highly endangered, and current management efforts include hybridisation with anadromous salmon populations (Eronen et al. 2021, 2023; Klemme et al. 2021, 2024b). Here, experimental crosses were produced using a 2 × 2 factorial design with 24 females and 24 males from each parental population, pairing one female and male from both LS and AS. This generated four crosses: LS × LS, AS × AS and their hybrids (LS × AS and AS × LS, female × male). After hatching, the crosses were kept in eight 3.14 m^2^ indoor tanks (two replicates per cross) for six months until they were tagged as described earlier. Subsequently, they were transferred to two 15 m^2^ indoor tanks, each housing 250 individuals from each cross. At the age of one year, 720 individuals (180 of each cross) were randomly selected for the experiment and measured for length (154.3 ± 14.2 mm) and mass (36.1 ± 9.0 g).

The Northern pike (*Esox lucius*) is a common natural predator of salmon. The individuals used in both experiments (N = 38) were wild caught from Lake Oulujärvi and maintained in large outdoor tanks. During the rearing period, all fish were fed with commercial fish food, and the water temperature as well as light cycle followed natural conditions.

### Experimental protocol, Experiment 1

**Fig. 1:**
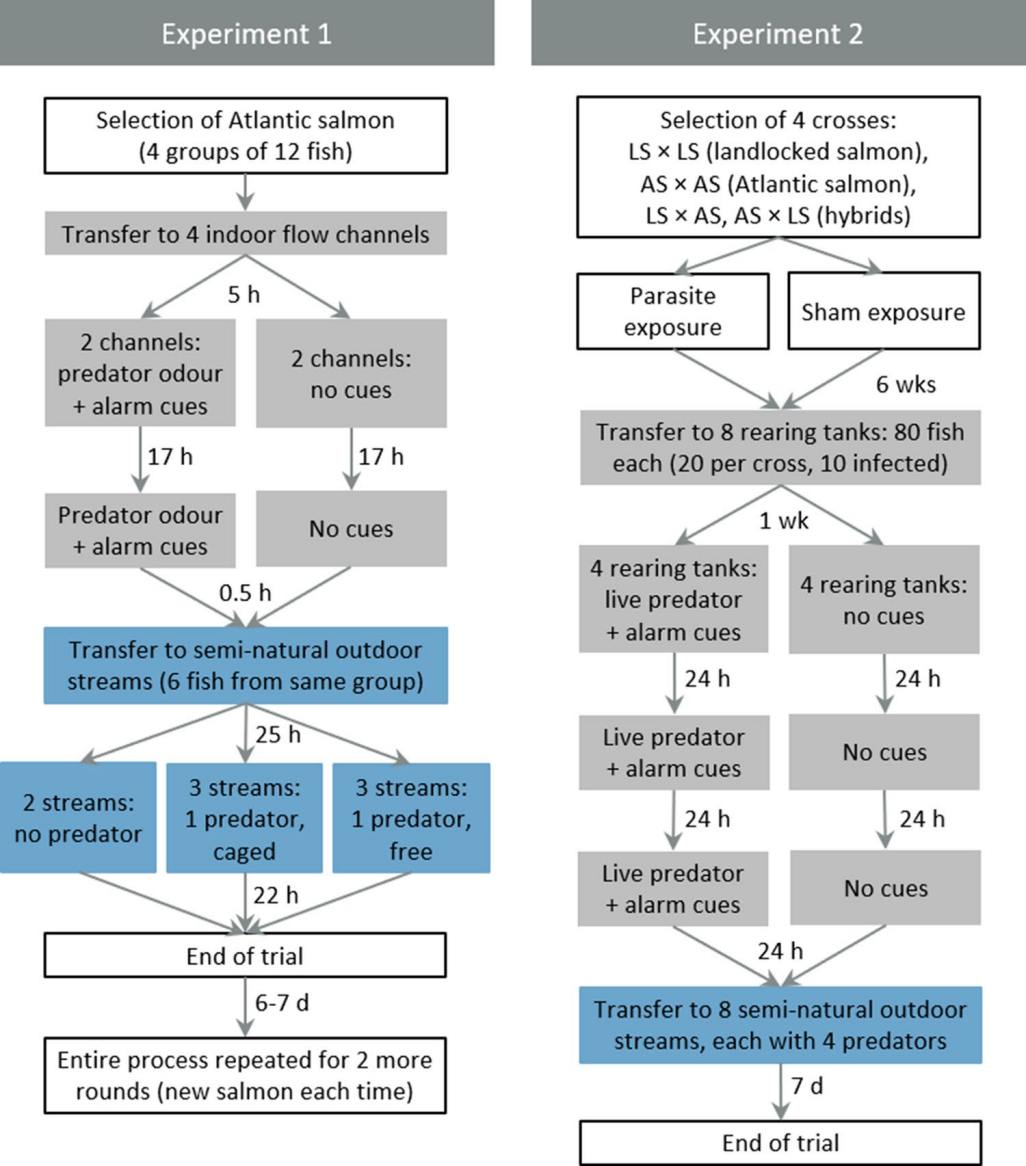
Flowchart showing the design and timeline of Experiment 1 and Experiment 2

To understand how antipredator training affects salmon movement behaviour and survival under predation risk, salmon were first exposed to predator odour and alarm cues from injured conspecifics during two training sessions. Salmon that were not exposed to these cues served as controls. A control group with pike odour only was not included because the aim was not to isolate learning mechanisms, but rather to evaluate whether a simple and easily implemented conditioning method can influence behaviour under ecologically realistic conditions. Following the training, salmon from both treatments were released to semi-natural outdoor streams with different predator treatments: no predator, caged predator, or free-swimming predator. Caged predators were used to prevent actual predation on the salmon, thus avoiding the release of alarm cues and ensuring complete behavioural profiles throughout the experiment. The study was conducted in three rounds, each separated by 6–7 days, using different groups of salmon in each round (Fig. 1).

For antipredator training, four groups of twelve salmon were each transferred to one indoor flow channel (length 6 m, width 0.4 m, water depth 0.15 m) with a gravel bottom. The channels were longitudinally divided into two compartments by wire mesh (mesh size 5 mm) about 2 m from one end. The smaller compartment, where the water outlet was located, contained the salmon, while the larger compartment with the water inflow (2–3 L s^−1^) was used to provide the cues. To avoid disturbance during cue administration, a black curtain shielded the experimenter. Predator odour was administrated upstream by directing water (1.5 L s^−1^) from a nearby tank with 3 pike (average body mass 2.4 ± 0.2 kg) into the channel. Alarm cues were administered by adding a mesh bag containing five freshly killed salmon (blow to the head followed by cervical dislocation) from the same population and cohort. Their skin was punctured along the entire body with thick needles to mimic injuries from a predator attack.

Two of the four channels were randomly assigned for antipredator training. Training began after five hours of acclimatisation in the training channels by switching the incoming water from clean to predator odour. After five minutes, alarm cues were introduced by placing the mesh bag into the channel between the incoming predator odour and the salmon compartment. Following an additional 5-min interval, the mesh bag was removed, and the incoming water reverted to clean. The two channels assigned to the control treatment remained untreated. Seventeen hours after the initial training session, the procedure was repeated. After a recovery period of 30 min, the salmon were transferred to eight semi-natural outdoor streams for the predation experiment.

**Fig. 2:**
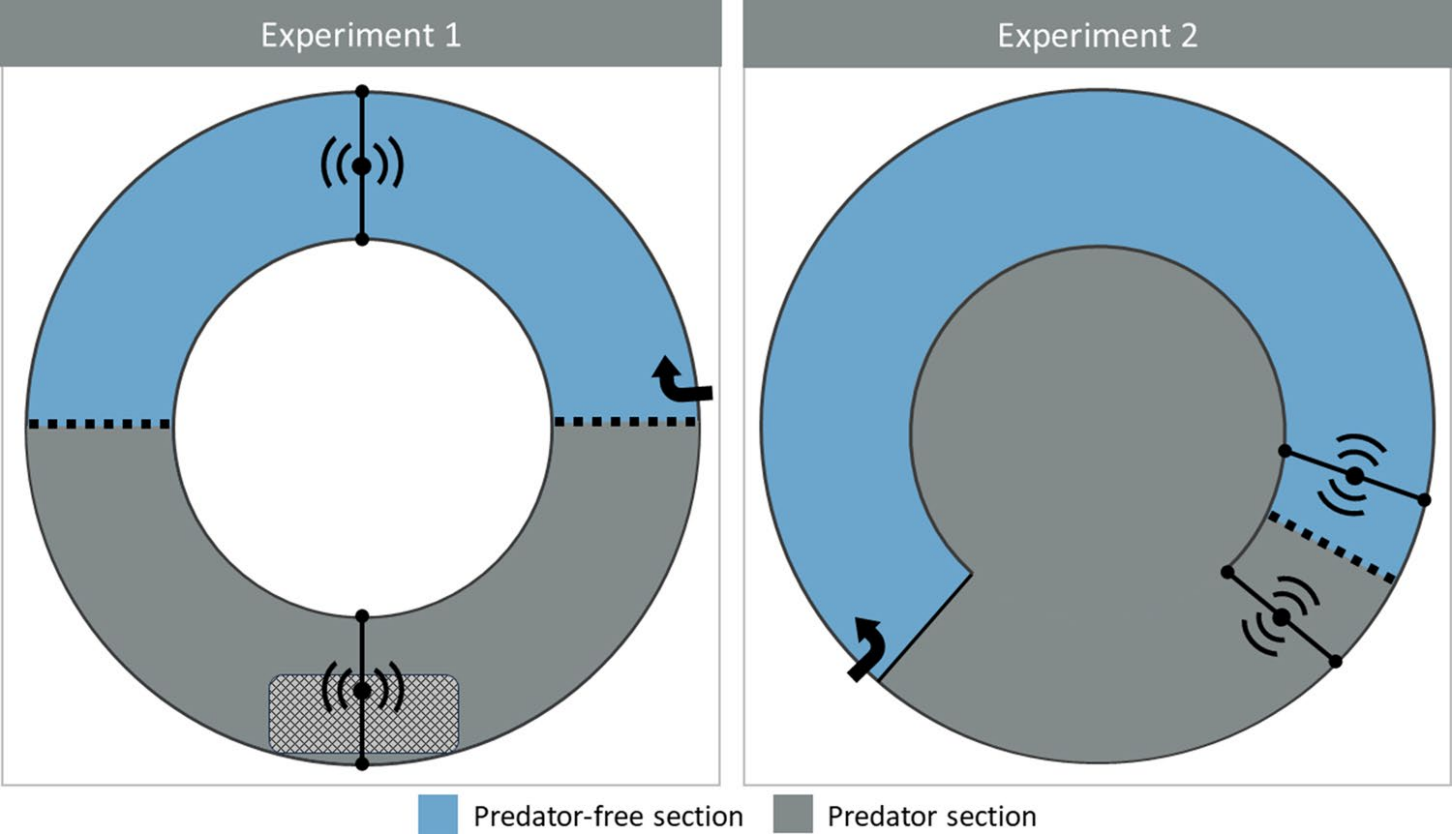
Layout of the semi-natural streams (from above) used for predation trials in Experiment 1 (N = 8) and Experiment 2 (N = 8). Streams were separated into a predator-free section and a predator section by wire mesh (dotted black line) that allowed salmon to pass but restricted pike movement. In each stream type, two RFID antennas (black lines with signal waves) recorded bypassing PIT-tagged salmon. Black arrows indicate the location and direction of the water inflow. In Experiment 1, some treatments included a mesh-cage (rectangle), which was positioned directly underneath the antenna in the predator section

The streams were ring-shaped with a surface area of 40 m^2^, a width of 1.5 m and length of 26.15 m in the middle (Fig. 2). The water depth was set to 30 cm with an induced current of 40 L s^−1^. The stream bed consisted of coarse gravel (ø 3–8 cm) interspersed with larger boulders (ø 15–30 cm, 1 boulder per m^2^) for shelter. To create distinct predator-free and predator sections, the streams were divided equally by two wire mesh walls (mesh size 5 cm). The mesh allowed salmon to move freely between both sections, while restricting free-swimming pike to the predator section. A PIT based radio frequency identification (RFID) antenna positioned in the middle of each section recorded the identity of passing PIT-tagged salmon at a rate of nine times per second, starting one hour after salmon release. This enabled monitoring salmon activity and spatial behaviour.

The experimental design used a 2 × 3 factorial setup with two antipredator training treatments (trained and control) and three predator exposure treatments (no predator, caged predator, and free predator). In each experimental round, there were four replicates for each training treatment. This included 2 replicates without predators (1 trained and 1 control), 3 replicates with a caged predator (either 2 trained and 1 control, or vice versa) and 3 replicates with a free-swimming predator (either 1 trained and 2 control, or vice versa). After three rounds, the total number of replicates was as follows: 3 trained without predator, 3 control without predator, 5 trained with a caged predator, 4 control with a caged predator, 4 trained with a free predator, and 5 control with a free predator.

Six salmon from the same training treatment were released into the predator-free section of each stream. Twenty-five hours later, the predator treatments were introduced to the predator section as follows: an empty cage for the ‘no predator’ replicates, a cage containing one pike for the ‘caged predator’ replicates, and an unrestricted pike for the ‘free predator’ replicates (average pike body mass 2.2 ± 0.4 kg). The cages measured 1.6 × 0.8 × 0.6 m and were made of wire mesh (mesh size 11 mm). They were placed on the stream bed next to the outer wall of the stream. The predation experiment lasted 22 h from the release of the pike. Afterward, the pike were returned to their maintenance tank, and the salmon were identified and euthanized using an overdose of Benzocaine (200 mg L^−1^). Between rounds, streams remained without fish for four days, with continuous water flow to minimize any residual chemical cues. Further, each stream retained the same predator treatment across rounds.

### Experimental protocol, Experiment 2

To test whether antipredator training enhances post-stocking survival and whether such learned predator avoidance is affected by genetic background or parasitic infection, salmon from either two purebred or two hybrid crosses were first exposed to eye parasites (control: sham exposure). They were then given antipredator training (control: no training) and finally released into semi-natural outdoor streams containing predators (Fig. 1).

To manipulate infection status, 360 fish (90 per cross) were exposed to the eye fluke *Diplostomum pseudospathaceum*, while another 360 fish received sham exposure. Outbreaks of *D. pseudospathaceum* are common in fish hatcheries (Stables and Chappell 1986; Field and Irwin 1994; Buchmann and Bresciani 1997; Karvonen et al. 2006). Here, infective stages (cercariae) were obtained from 20 naturally infected *Lymnaea stagnalis* snails (first intermediate host) collected from the wild. The snails were placed in individual containers with lake water at room temperature for cercarial shedding. After four hours, the average cercarial density in the combined solution of all snails was estimated using ten 1 ml aliquots.

For the exposure, the fish were evenly distributed among sixteen 80 L containers (N = 45 fish per container) filled with aerated lake water (15.4 °C). Half of the containers received 15,750 cercariae (350 per fish) in a small amount of lake water, while the other half received only lake water. After 30 min of exposure, the fish were transferred to two mixed-treatment 3.2 m^2^ tanks. Infection in the eye or its absence in the control treatment was confirmed five weeks post-exposure using slit lamp microscopy (Kowa SL-15) under mild anaesthesia (Benzocaine, 40 mg L^−1^). The parasite induces eye cataracts (Karvonen 2012) and the proportion of eye lens covered by the cataracts was assessed as 0–1 in increments of 0.1. At the same time, fish body mass and length were measured, and 320 infected and 320 uninfected individuals were sorted into eight experimental groups. Each group consisted of 80 fish, 20 of each cross, with 10 being infected. The groups were individually maintained in 3.2 m^2^ tanks, which were used for antipredator training.

Antipredator training was conducted six weeks post exposure. Four experimental groups were exposed to predator and alarm cues, while the other four groups served as control. For both treatments, a cage was installed in the rearing tanks 24 h before the training commenced. The cages (80 × 60 × 40 cm) were made of hard plastic, with the two shorter ends (60 × 40 cm) constructed from wire mesh (mesh size 5 mm). Immediately before the training, the water inflow was set to 0.2 L s^−1^ in all tanks. The alarm cues were prepared as described above, using four salmon, one of each cross (average length: 154.3 ± 15.7 mm), for each experimental group undergoing training. The dead salmon were added together with one live pike (1–2 kg) to the cage. After 10 min of exposure, the pike and dead salmon were removed, and the water inflow reset to 1 L s^−1^. In the control tanks, the addition and removal of pike and alarm cues were simulated using a clean dip net, but no actual cues were added. The training procedure was conducted three times at 24-h intervals.

Twenty-four hours after the last training session, the experimental groups were released into eight semi-natural outdoor streams (Fig. 2) to monitor their survival under pike predation. The round streams (50 m^2^) were divided in two sections: a predator-free section at the outer edge (15 m^2^) and a predator section in the middle (35 m^2^). The outer section resembled a shallow river area with a water depth of 22 cm, a moderate water flow of 4 L s^−1^, and a stream bed with coarse gravel (ø 5–7 cm) and larger stones (ø 20 cm). The middle section mimicked deeper river areas (water depth 78 cm) with slowly flowing to stagnant water and no structures. The sections were separated with a C-shaped wall, closed at one end, and fitted with a wire mesh (mesh size 50 mm) at the other. The mesh allowed salmon to pass, but restricted pike to the predator section. On either side of the mesh, a PIT-based radio frequency identification antenna recorded the identity of passing salmon to monitor their movement between the predator-free and predator section.

Each predator section of the eight streams held four pike (1.8 ± 0.5 kg), which were released one week before the experiment for acclimatisation. The eight experimental salmon groups (either trained or control) were each released to one predator-free section. Initially, the wire mesh opening between the stream sections was blocked, and then opened one hour after the salmon had been released. During the experiment, salmon relied on natural food resources, consisting of benthic and drifting invertebrates. The streams were left undisturbed for seven days, after which all remaining salmon were identified and euthanized with an overdose of benzocaine (200 mg L^−1^). The pike were returned to their maintenance tank.

### Data analysis

For Experiment 1, two variables were extracted from the RFID data: activity and proportion of time spent in each subsection, measured over two 22-h periods (10:00 am–8:00 am), before and after the predator treatment commenced. Mortality in replicates with free predators was low (5/54 salmon; 2 trained and 3 control) and therefore not formally analysed. Because the depredated individuals had incomplete RFID data, they were removed from the dataset, leaving the total sample size at N = 139.

For activity, the total number of completed half-rounds – defined as consecutive detections at both antennas – was extracted. Activity was analysed with a generalized linear mixed model with negative binomial error distribution and log-link. The fixed effects were period (before or after the predator treatment commenced), antipredator training treatment (control or trained) and predator treatment (no predator, caged predator, or free predator). Random effects were incorporated to account for pseudo-replication and potential spatial–temporal variation, using salmon identity, stream identity and conditioning group nested within experimental round.

To determine the proportion of time spent in each subsection, an individual binary variable (recorded/not recorded) was extracted for each antenna and each minute. If no recordings were made during a minute at either antenna, it was assumed that the salmon remained in the section where it was last recorded. From these, the proportion of time spent in the predator section was extracted for each minute (0, 0.5 or 1), and the average proportion over time was calculated. This variable was then included as response variable in a generalized linear mixed model with beta error distribution and logit-link. The model was fitted with the same fixed and random effects as described above.

In Experiment 2, three variables were analysed: mortality, the probability of entering the predator section, and eye cataract coverage among infected salmon. Two replicates (one control and one trained) were excluded from the analysis due to a malfunctioning antenna at the entrance to the predator section. Additionally, nine fish from the remaining replicates were removed from the mortality analysis (total N = 471) and six fish from the entering probability analysis (N = 474) due to incomplete RFID data.

Mortality was analysed using a mixed-effects Cox model, with mortality as the event and time to death (last observation at the antenna in the predator section) as the time variable. Fixed effects included antipredator training treatment, infection treatment, and cross, while stream identity was included as a random effect. As all fixed-effect interactions were non-significant, a reduced model without interactions was fit and a likelihood ratio test was used to compare both models. The model including the interactions did not show a significantly better fit (*Χ*^2^ = 8.3, *df* = 10, *p* = 0.600) and the reduced model was selected.

The probability of entering the predator section was also assessed using a mixed-effects Cox model. In this model, entry into the predator section was treated as the event, and the time to entry (first observation at the antenna in the predator section) was used as the time variable. The same fixed and random effects as described for mortality were applied.

Variation in eye cataract coverage was analysed for all salmon from the infection treatment using a generalised linear model with beta error distribution and logit-link, and cross as fixed factor.

All models were run in R, version 4.4.0 (R Core Team 2020), using the ‘TMB package’ (Kristensen et al. 2016), the ‘coxme package’ (Therneau 1997) and the ‘emmeans package’ (Lenth and Lenth 2018). The significance values of post-hoc comparisons were adjusted using the Tukey method. Comparisons were based on estimated marginal means from the fitted model, with significance assessed using Wald z-tests derived from model-based contrasts and their standard errors. Hazard ratios (HR) are presented with their standard errors.

## Results

### Experiment 1

**Fig. 3:**
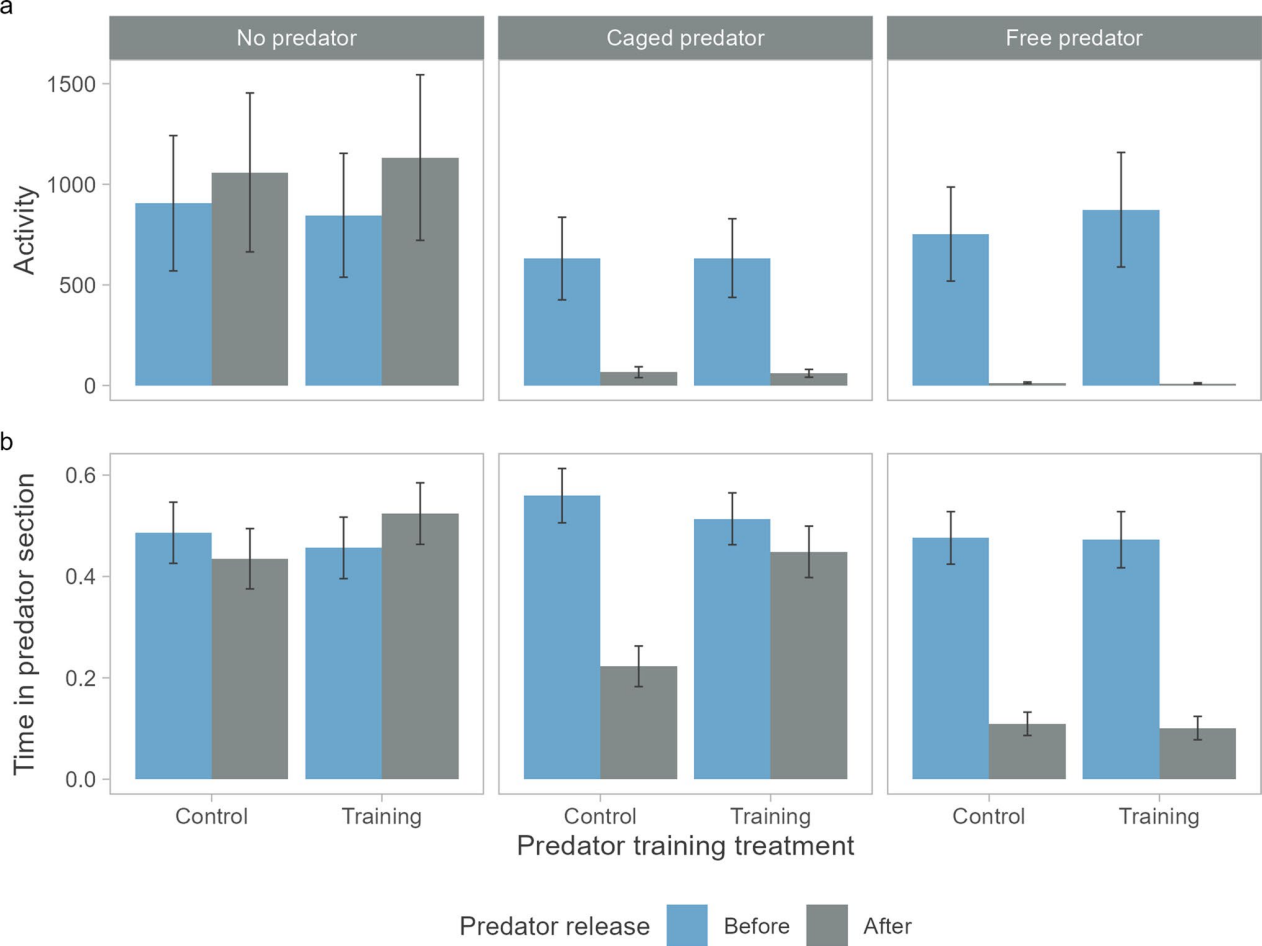
Predicted means (± SE) of **a** salmon activity (total number of half rounds, N = 139) and **b**average proportion of time spent in the predator section in seminatural streams before and after the addition of a pike predator (N = 139) in Experiment 1. The predator was either absent (N = 6 replicates), caged (predation prevented, N = 9 replicates) or free-swimming within the predator section (predation possible, N = 9 replicates). Before release to the streams, salmon underwent antipredator training, i.e., they were either exposed to predator odours and alarm substances (trained, N = 70) or were not exposed (control, N = 69)

Salmon activity was influenced by an interaction of period (before/after predator release) with predator treatment (Table 1, Fig. 3a). In streams without predators, there was no significant change in salmon activity between the two periods (*z* = 1.3, *P* = 0.199). However, in streams with predators, salmon reduced their activity after the introduction of the predators (cage: *z* = −10.3, *P* < 0.001, free: *z* = −25.9, *P* < 0.001). This reduction in activity was more pronounced in streams with free predators (98.6%) than in those with caged predators (90.0%) and both treatments differed significantly in activity after the introduction of the predators (*z* = 5.2, *P* < 0.001). Antipredator training had no effect on salmon activity (Table 1).

**Table 1:** Fixed parameter estimates for two generalized linear mixed models on salmon activity and proportion of time spent in the predator section in Experiment 1 (N = 139)

	Activity			Time in predator section		
Parameter	Wald *Χ*^*2*^	*df*	*P*	Wald *Χ*^*2*^	*df*	*P*
Period	0.4	1	0.524	0.8	1	0.361
Predator treatment	0.8	2	0.670	2.3	2	0.322
Antipredator training	< 0.1	1	0.852	0.2	1	0.686
Period:treatment	177.0	2	< 0.001	34.6	2	< 0.001
Period:training	0.2	1	0.696	2.2	1	0.136
Period:treatment:training	1.4	4	0.840	12.4	4	0.015

The proportion of time spent in the predator section was influenced by a three-way interaction among period, predator treatment, and antipredator training treatment (Table 1, Fig. 3b), as the effect of period depended on both the predator and training treatments. Specifically, in replicates without predators, the time spent in the predator section did not differ between periods for either training treatment (control: *z* = −0.9, *P* = 0.361; trained *z* = 1.2, *P* = 0.232). In replicates with caged pike, the time spent in the predator section decreased after predator release, but this was only significant for control salmon (control: 77.4% reduction, *z* = −7.0, *P* < 0.001; trained: 23.0%, *z* = −1.5, *P* = 0.137). In replicates with free pike, the time spent in the predator section decreased significantly for both training treatments (control: 86.5%, *z* = −9.3, *P* < 0.001; trained: 87.5%, *z* = −8.6, *P* < 0.001). The proportion of time spent in the predator section after predator release was lower in streams with free pike than in streams with caged pike in both training treatments (control: *z* = 3.1, *P* = 0.006; trained: *z* = 7.4, *P* < 0.001). Consequently, the only significant training treatment contrast was found for replicates with caged pike after predator release (*z* = 4.0, *P* < 0.001, all other *P* > 0.228).

### Experiment 2

**Fig. 4:**
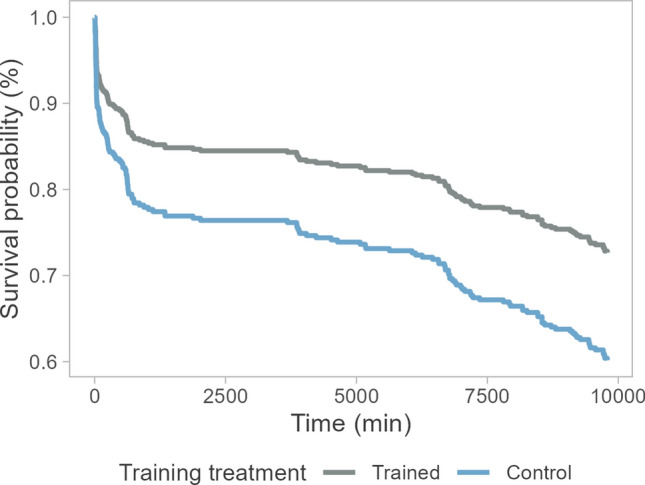
Predicted survival curves for salmon in semi-natural streams with pike predators in Experiment 2 (N = 471). Before release to the streams, salmon underwent antipredator training, i.e., they were either exposed to predator odours and alarm substances (trained, N = 235) or not exposed (control, N = 236)

During the predation experiment, 161/472 salmon were taken by the predators. Mortality was significantly influenced by antipredator training (*Χ*^*2*^ = 5.2, df = 1, *P* = 0.023), with trained salmon showing a 38% lower risk of mortality than control salmon (HR 0.62 ± 0.21, Fig. 4). Infection treatment had a marginally significant effect (*Χ*^*2*^ = 3.1, df = 1, *P* = 0.077), with infected salmon exhibiting a 33% higher mortality risk (HR 1.33 ± 0.16).

The average proportion of eye lens covered by parasite-induced cataracts was 0.31 ± 0.15. Cross had a marginally significant effect on cataract coverage (Wald *Χ*^*2*^ = 7.5, df = 3, *P* = 0.057), with purebred AS having the highest coverage (LS × LS: 0.30, AS × AS: 0.35, LS × AS: 0.31, AS × LS: 0.29). However, there were no significant differences in mortality among the salmon crosses (*Χ*^*2*^ = 5.9, df = 3, *P* = 0.119).

**Fig. 5:**
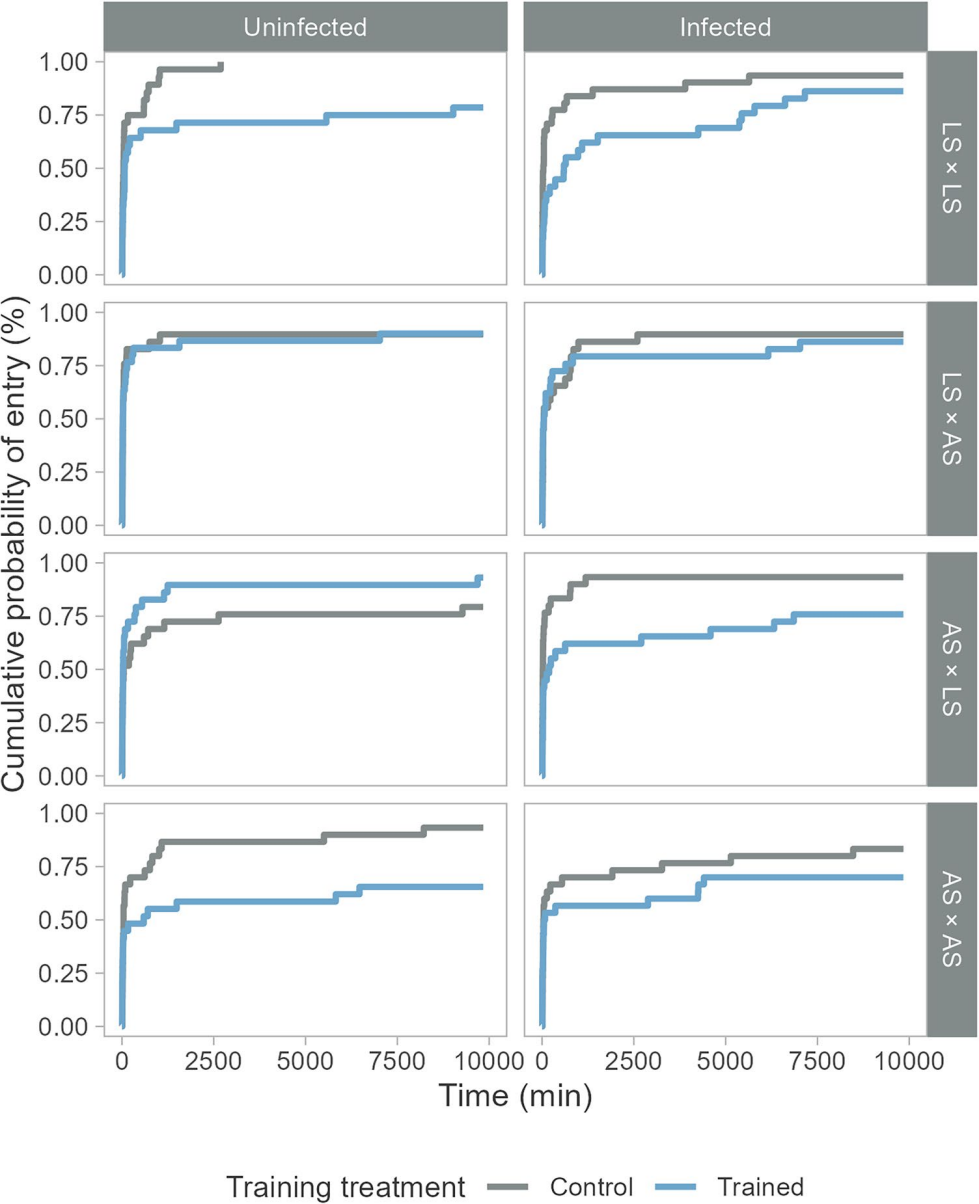
The probability of salmon to enter the predator section in semi-natural streams in Experiment 2 (N = 474). Salmon originated from one of four experimental crosses, landlocked salmon (LS × LS, N = 118), Atlantic salmon (AS × AS, N = 120) or their hybrids (LS × AS, N = 118 and AS × LS, N = 118; female × male). Before release to the streams, salmon were either exposed to the eye fluke *D. pseudospathaceum* (infected, N = 240) or sham exposed (uninfected, N = 234). Subsequently, they underwent antipredator training, i.e., they were either exposed to predator odours and alarm substances (trained, N = 236) or were not exposed (control, N = 238)

The effect of antipredator training treatment on the probability of entering the predator section depended on the interaction between infection treatment and cross (Table 2). In most antipredator training contrasts, trained salmon had a lower probability of entering the predator section than control salmon (overall 45%, HR 0.55 ± 0.43). In two contrasts among uninfected crosses, however, this pattern was either reversed (AS × LS) or both treatments had equal probability (LS × AS, Fig. 5).

**Table 2:** Fixed parameter estimates of a mixed-effects Cox model on the probability of entering the predator section in Experiment 2 (N = 474)

Parameter	*Χ*^*2*^	*df*	*P*
Antipredator training	1.9	1	0.164
Infection	0.1	1	0.771
Cross	4.2	3	0.245
Training:infection	< 0.1	1	0.940
Training:cross	10.5	3	0.015
Infection:cross	7.5	3	0.057
Training:infection:cross	8.7	3	0.034

## Discussion

Predation is generally considered the main reason for failures of conservation programmes that involve the release of captive-reared animals to augment or restore populations (Berejikian 1995; Henderson and Letcher 2003; Moseby et al. 2011). Our experiments demonstrate that conditioning captive-reared salmon with predator cues before release can significantly enhance post-release survival, but also that the effectiveness of such antipredator training is context dependent. These findings are consistent with previous studies suggesting that antipredator learning and memory are complex processes (reviewed in Brown et al. 2013; Edwards et al. 2021; Zhu et al. 2023). Notably, parasite infections and genetic background did not affect the outcomes of antipredator training, suggesting that these factors are of minor importance, at least in the present system. Overall, our results advance the understanding of the effectiveness of antipredator training and provide methods to improve programmes aimed at sustaining and reintroducing natural Atlantic salmon populations.

### Experiment 1

In the first experiment, salmon exhibited clear innate antipredator behaviour towards pike as individuals without prior antipredator training showed reduced activity in the presence of pike and avoided the stream section with the predator. This confirms that Atlantic salmon retain the innate recognition of pike, previously observed in 0 + juveniles (Hawkins et al. 2008), over extended periods of captive rearing (2 + years). The antipredator response was stronger when pike were free-swimming than when caged. This may be explained by more variable predator cues, as salmon may have perceived primarily olfactory cues from caged pike while free-swimming pike likely also provided visual, auditory and mechanosensory cues. In addition, predation by free-swimming pike could have released alarm cues that intensified the response. However, this explanation is less likely given the low level of predation, observed in only 4 of 9 replicates.

Innate predator responses of salmonids can be enhanced by antipredator training (Berejikian et al. 2003), but this effect was not observed in Experiment 1. Both trained and untrained salmon showed similar activity levels in the presence of caged and free-swimming pike, and similar spatial avoidance of free pike. Chemical conditioning followed by exposure to live predators, providing both visual and chemical cues, has been previously used in studies aiming to improve post-release survival of hatchery-reared fish (Berejikian et al. 1999; Mirza and Chivers 2000; Gazdewich and Chivers 2002). This approach is particularly relevant because it allows testing for behavioural responses under ecologically realistic conditions, while using a low-cost training method that is easy to implement in hatchery settings (Berejikian et al. 2003). In our study, however, the visual cues from the live predator may have elicited robust innate responses in both treatment groups, potentially masking any behavioural differences resulting from prior chemical conditioning.

Surprisingly, trained salmon did not spatially avoid caged pike as opposed to untrained salmon. One possible explanation is habituation to predator odour from repeated training events, which can weaken the antipredator response (Brown et al. 2013). In fish, there is evidence that older individuals (here 2 + years), who typically face lower predation risk than younger conspecifics, could habituate faster to predator stimuli (Berejikian et al. 2003; Vilhunen 2006). Nevertheless, trained salmon responded to the presence of caged pike by reducing their activity. Therefore, it is possible that the training conditions elicited these responses. Training was conducted in small tanks (0.16 m^2^), which may have promoted reduced activity in response to predation risk instead of spatial avoidance. However, trained salmon did avoid the predator section when pike were free-swimming, indicating that any potential issues associated with the training method did not result in maladaptive responses under actual predation risk.

The low predation-induced mortality did not allow assessment of actual survival benefits of antipredator training. Since both trained and control salmon showed similar behaviours, it is unlikely that survival differences would have emerged even with a higher predation rate. However, possible differences in other behaviours than those observed here, such as vigilance, cannot be ruled out. The low observed mortality may be attributed to several factors, including the low prey density, which lowered the likelihood of predator–prey encounters. The duration of the predation trials was also short (22 h), although most post-release mortality in the wild typically occurs immediately after release (Olla et al. 1998, see also Fig. 4). Further, the pike were not acclimated to the stream environment before the predation trials, which may have reduced their propensity to feed.

### Experiment 2

In the second experiment, antipredator training increased post-release survival, likely because trained salmon were better able to avoid predators spatially. Trained individuals had a 45% lower probability of entering the stream section with the predators, though this variable varied among the salmon crosses and with the parasite infection status. Studying actual survival benefits of antipredator training under natural conditions, rather than just behavioural changes in the laboratory is crucial, as animals face multiple stressors during the release procedure that may mute learned behaviours (Brown et al. 2013; Zhu et al. 2023). By releasing salmon into semi-natural outdoor streams, we simulated the main stressors of stocking events, including handling, transport, changes in environmental conditions, and transition to feeding on live prey. Thus, our study provides rare evidence of post-release survival benefits from antipredator training. Although substantial research has focused on antipredator training in fish (reviewed in Brown et al. 2013; Näslund 2021), largely due to the importance of stocking as a fishery management tool, we found only four studies that evaluated the survival benefits of this method in semi-natural or natural conditions. Of these, two studies demonstrated increased post-stocking survival (Mirza and Chivers 2000; D’Anna et al. 2012), while the other two reported no difference between trained and control fish (Berejikian et al. 1999; Hawkins et al. 2007). On a broader scale, meta-analyses across multiple taxonomic groups (invertebrates, fish, birds, and mammals) have also shown positive survival effects from pre-release antipredator training (Tetzlaff et al. 2019; Zhu et al. 2023). However, the number of studies included was limited, suggesting that more research is needed to corroborate the findings.

Similar to previous studies, our two experiments provided mixed results on the effectiveness of antipredator training, suggesting that the success of this method is context dependent. Several factors, primarily differences in experimental protocols, may have contributed to the varying outcomes. First, the training tanks differed considerably in size (0.16 *vs.* 3.2 m^2^) and shape, potentially influencing the behaviours learned, as discussed above. Second, the strength and type of predator cues likely differed: Experiment 1 used odours from a predator tank in two training sessions in flow-through channels, while Experiment 2 involved a live (caged) predator in three training sessions with reduced water flow. Higher concentrations of predator odour (although not quantified in this study) and more frequent training sessions have been shown to strengthen conditioning (Ferrari et al. 2005; Zhao et al. 2006; Vilhunen 2006). Third, the predation experiments differed in predator density (one pike in Experiment 1 vs. three in Experiment 2) and stream layout, both of which may have influenced salmon behaviour. Finally, the salmon differed in age and developmental stage, with two-year-old smolts (migrating juveniles) used in Experiment 1 and one-year-old parr (not yet migrating juveniles) in Experiment 2. Age-specific propensities for antipredator learning have been demonstrated in Atlantic salmon, though previous studies focused on juveniles aged 3–36 weeks (Hawkins et al. 2008). Additionally, both learning and physiological development are energetically demanding processes and the smoltification (transition from fresh- to sea water-adapted juveniles) undergone by the salmon shortly before Experiment 1 may have reduced resources available for learning (Brown et al. 2013). This aligns with earlier findings in Atlantic salmon, where no survival benefits of antipredator training were observed during migration (Hawkins et al. 2007).

Constraints on learning ability and cue reception can also arise from parasitic infections and their associated energy costs (Binning et al. 2018; Reichert et al. 2023). However, we found no evidence that infection with *D. pseudospathaceum* affected antipredator learning, as both uninfected and infected salmon showed improved survival following training. *Diplostomum pseudospathaceum* infects the eye lenses of fish, causing cataracts that interfere with food detection (Karvonen and Seppälä 2008; Klemme et al. 2024b) and subsequently reduce energy levels. Despite this, infected salmon may prioritize learning about predators, as cataracts also increase predation susceptibility (Seppälä et al. 2005; Klemme et al. 2024b). Besides draining host energy, parasites can also impair antipredator learning by damaging organs critical for cue reception or processing (Reichert et al. 2023). Future studies could therefore explore infections of olfactory organs and the central nervous system when assessing the role of parasitic infections on antipredator learning.

Hybridisation also had no effect on antipredator learning, as training improved survival in both purebred and hybrid crosses. Juveniles from both parental populations inhabit rivers before migrating either to a lake (landlocked salmon) or to the sea (anadromous salmon), facing similar risks of predation during this life stage. As a result, the likelihood of intermediate phenotypes with different predator avoidance strategies is reduced. However, the effect of antipredator training on avoiding the predator section was less clear in hybrids compared to purebred crosses, particularly among uninfected individuals, though this did not result in detectable survival differences. Generally, whether hybrids exhibit maladaptive learning remains an understudied question in conservation research (Rice and McQuillan 2018).

In addition to antipredator training, other strategies have been explored to improve the post-release success of hatchery-reared salmon. These include environmental enrichment during rearing, which facilitates the development of more natural foraging and risk-assessment behaviours, and soft-release protocols that provide fish with a gradual transition to the wild (Brown and Day 2002). These studies have shown that such measures can enhance foraging efficiency (Brown et al. 2003; Rodewald et al. 2011), reduce stress responses (Näslund et al. 2013; Roberts et al. 2024), and increase survival under natural predation pressure (Hyvärinen and Rodewald 2013). Integrating these approaches with targeted antipredator training may offer a more comprehensive strategy for improving reintroduction outcomes.

## Conclusions

Atlantic salmon is an important species with large socioeconomic impact and over 5 billion captive-reared individuals are released worldwide every year (Brown and Day 2002). We show that antipredator training, when conducted under certain conditions, can increase the post-release survival of Atlantic salmon. Notably, training was effective when conducted within rearing tanks, suggesting that large-scale antipredator training could be easily implemented in hatcheries. This may provide a cost-effective method for improving the success of population enhancement strategies. The possibility that smoltification could constrain learning requires further study, particularly as this life stage, at which salmon prepare to enter the marine environment, is commonly used for stocking (Verspoor et al. 2007). More generally, additional research is needed to understand potential limitations of antipredator training arising from parasite infections acquired in captivity and from hybridisation protocols used for conservation. While our findings have important implications for restocking programmes of Atlantic salmon, they may also be applicable to recovery programmes of other highly endangered species, for which experimental testing of antipredator training is not possible.

## Data Availability

All data supporting the findings of this study are available on Zenodo (Klemme et al. 2024a).
